# Design of Mantis-Shrimp-Inspired Multifunctional Imaging Sensors with Simultaneous Spectrum and Polarization Detection Capability at a Wide Waveband

**DOI:** 10.3390/s24051689

**Published:** 2024-03-06

**Authors:** Tianxin Wang, Shuai Wang, Bo Gao, Chenxi Li, Weixing Yu

**Affiliations:** 1 Key Laboratory of Spectral Imaging Technology, Xi’an Institute of Optics and Precision Mechanics, Chinese Academy of Sciences, Xi’an 710119, China; wangtianxin@opt.ac.cn (T.W.); gaobo_101@opt.ac.cn (B.G.); lichenxi@opt.ac.cn (C.L.); 2 Center of Materials Science and Optoelectronics Engineering, University of Chinese Academy of Sciences, Beijing 100049, China

**Keywords:** mantis shrimp, Fabry–Pérot resonator, multispectral, polarization

## Abstract

The remarkable light perception abilities of the mantis shrimp, which span a broad spectrum ranging from 300 nm to 720 nm and include the detection of polarized light, serve as the inspiration for our exploration. Drawing insights from the mantis shrimp’s unique visual system, we propose the design of a multifunctional imaging sensor capable of concurrently detecting spectrum and polarization across a wide waveband. This sensor is able to show spectral imaging capability through the utilization of a 16-channel multi-waveband Fabry–Pérot (FP) resonator filter array. The design incorporates a composite thin film structure comprising metal and dielectric layers as the reflector of the resonant cavity. The resulting metal–dielectric composite film FP resonator extends the operating bandwidth to cover both visible and infrared regions, specifically spanning a broader range from 450 nm to 900 nm. Furthermore, within this operational bandwidth, the metal–dielectric composite film FP resonator demonstrates an average peak transmittance exceeding 60%, representing a notable improvement over the metallic resonator. Additionally, aluminum-based metallic grating arrays are incorporated beneath the FP filter array to capture polarization information. This innovative approach enables the simultaneous acquisition of spectrum and polarization information using a single sensor device. The outcomes of this research hold promise for advancing the development of high-performance, multifunctional optical sensors, thereby unlocking new possibilities in the field of optical information acquisition.

## 1. Introduction

When you gaze deeply at an animal, it gazes back at you. Over millions of years of evolution, the visual systems of all living organisms have developed distinct characteristics. As scientific knowledge deepens and technology advances, our understanding of the visual mechanisms in other species continues to expand [1,2,3,4,5]. Simultaneously, we are increasingly appreciating the unique advantages inherent in these biological systems. Motivated by insights from biological vision, a range of bioinspired vision technologies [6,7,8], including compound eye cameras [9,10], fish-eye cameras [11], cat-eye cameras [12], and snake-eye cameras [13], are being progressively employed across diverse contexts to meet specific requirements. For instance, fish-eye cameras provide imaging with an expansive 180° field of view, facilitating panoramic imagery [14]. Taking cues from the compound eyes of insects, bioinspired compound eye cameras utilize multiple independent imaging units to establish a comprehensive imaging system, enabling wide-field-of-view imaging and the precise detection and tracking of moving objects [15,16,17].

The mantis shrimp is renowned for having the most complex visual system discovered to date. It possesses 16 unique types of photoreceptor cells that can detect light within a wavelength range of 300–720 nm, including various polarizations [18,19]. This extraordinary visual system has inspired significant advancements in bioinspired imaging technology [20,21]. Unlike the human brain, which discerns colors by processing relative signal strengths from three types of cone cells, the mantis shrimp’s visual system employs 16 finely tuned cone cells. This suggests a more efficient process, resulting in a richer acquisition of target information with fewer cognitive processes. This alignment of processes is consistent with the design philosophy of snapshot multispectral imaging devices. As an adept underwater predator, the mantis shrimp uses polarization information to help identify prey in complex underwater environments. Studies have shown that the use of polarized light can increase imaging distances underwater [22]. Drawing inspiration from the mantis shrimp’s sophisticated visual system, which uses multiple cone cells to simultaneously detect polarized information and identify targets, a kind of composite multifunctional apparatus has been developed. This apparatus integrates spectral detection devices with polarization devices, demonstrating its unique advantages in various complex application environments. Polarization cameras, developed based on this principle, have potential applications in areas such as tumor detection [23,24] and underwater imaging [25].

Integrating complex functionalities such as a broad wavelength range, high spectral resolution, and polarization detection into a single imaging device results in a bulky and structurally complex apparatus [26]. However, the advent of on-chip imaging technology enables the miniaturization of devices while still achieving multiple functionalities [27]. For polarization detection in visible light, a simple metal grating structure is adequate for polarization filtering [28]. Traditional Fabry–Pérot (FP) resonators and emerging metasurface filter devices can be integrated with complementary metal–oxide–semiconductor (CMOS) devices. This integration allows for the periodic arrangement of filter units on CMOS devices, thereby enabling multispectral imaging [29,30,31]. FP resonators, compared to metasurface filter devices, have a simpler structure, and do not depend on complex spectral reconstruction algorithms. Multispectral imaging devices, including wedge-shaped resonators and multi-step etched array resonators, have already made their mark in the market [32,33,34]. However, the limited high reflectance bandwidth of the reflectors that make up the FP resonator hinders the further expansion of the operational bandwidth of the FP resonator cavity. Whether using dielectric Bragg gratings or metal as resonator reflectors, achieving complete coverage of visible light and partial near-infrared (NIR) light while maintaining a working bandwidth above 400 nm and high transmission efficiency simultaneously poses a significant challenge.

In this paper, we introduce a novel reflector structure comprising a thin film system composed of a metal film pair and a high-refractive-index dielectric film. The resonator formed by this composite film system achieves high transmission efficiency in the 450–900 nm range. Building upon this, we develop a 16-channel narrowband FP resonator filter array utilizing this designed resonator structure. Furthermore, we integrate a polarization filtering grating beneath the filter array, allowing for polarization detection alongside multispectral imaging. Inspired by the mantis shrimp’s visual system, this structure facilitates wide-bandwidth multispectral imaging and polarization detection, with the potential for integration with CMOS devices.

## 2. Design and Result

**Figure 1:**
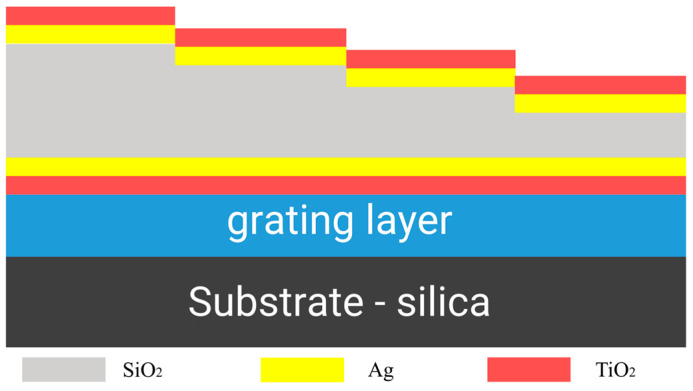
Structural schematic diagram of multifunctional imaging sensors.

Snapshot spectral imaging systems acquire multiple spectral data within a single detector integration period, requiring the design of multiple spectral channels within the same detector plane. To achieve the identification of polarization information while detecting spectral information, polarization detection devices need to be added to the incident light path. The center wavelength of the transmission band of the FP cavity can be adjusted by altering the resonator cavity length *h*, as indicated by the following formula: *λ* = 2*nhcosθ*/*k* (*k* = 0, 1, 2…). This design results in structures of varying heights by varying the thickness of the resonator cavity to correspond to different channel positions. To mitigate the impact of uneven surfaces on the polarization device, we considered placing the polarization device in an intermediate layer, as illustrated in Figure 1.

### 2.1. Design of Metal–Dielectric Composite Film Filter

The peak transmission and full width at half maximum (FWHM) of the transmission spectra in the FP resonator filter are determined by the parameters of the reflective mirrors forming the resonator cavity. The maximum transmission efficiency of the resonator filter is expressed as follows [35]:

(1) $$T_{\max}=\frac{T}{{\left(1-R\right)}^{2}}=\frac{T}{{\left(T+A\right)}^{2}}$$

**Figure 2:**
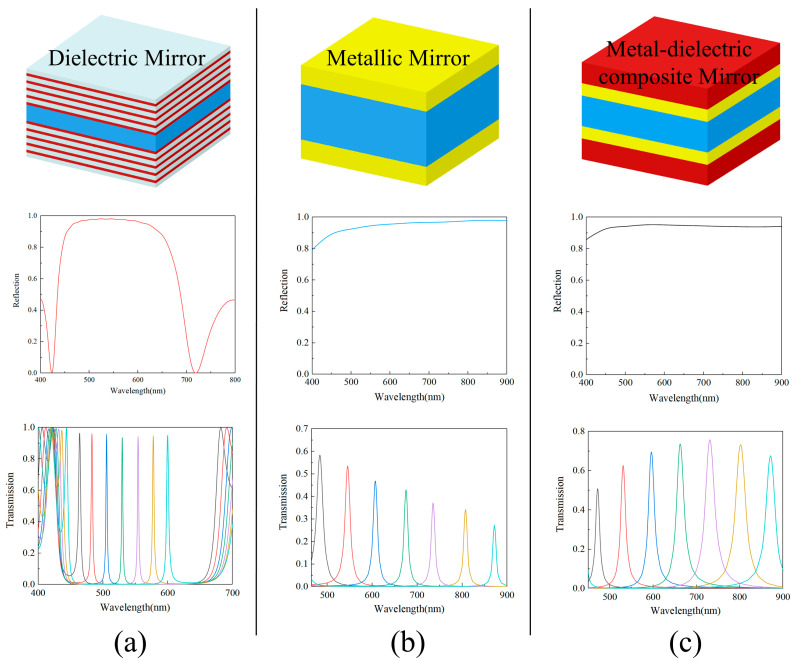
Comparison of reflectance efficiency of three types of reflectors and simulated transmission spectra of resonators (red material represents TiO_2_, yellow material represents Ag, white material represents SiO_2_, and blue represents resonators with variable height): (**a**) simulation results of a filter composed of all-dielectric reflectors with a central wavelength of 530 nm; (**b**) simulation results of a filter composed of 40 nm thick metallic silver; (**c**) simulation results of a metallic–dielectric composite film filter composed of 40 nm thick metallic silver and 68 nm thick TiO_2_.

Here, *T* represents the transmission efficiency of the reflective mirror, *R* is the reflection efficiency, and *A* is the absorption efficiency. Governing the spectral resolution, the FWHM is expressed as follows [33]:

(2) $$\mathrm{FHWM}=\frac{2\lambda_{0}}{k\pi }\arcsin(\frac{1-R-A}{2\sqrt{R}})$$

where *λ*_0_ is the peak wavelength of the transmission spectra and *k* is the interference order (*k* = 0, 1, 2…). It is evident that the transmission and FWHM of the FP filter are related to the efficiency of reflection and transmission of the reflective mirror of the given material. Dielectric reflective mirrors, which exhibit high reflection without significant absorption effects, result in higher transmission and a narrower full width at half maximum (FWHM) within the operating bandwidth of the Fabry–Pérot (FP) filter. However, the operational bandwidth of the FP filter is limited by the narrow high reflection band of the Bragg grating, as shown in Figure 2a. In contrast, metallic reflective mirrors provide higher reflection efficiency and a broader high reflection band. However, they experience reduced transmission in the long-wavelength region due to the metal’s absorption effects, as illustrated in Figure 2b. We developed a novel metallic–dielectric composite film filter, depicted in Figure 2c. Compared to existing resonators that use either metallic or dielectric materials as reflectors, our design addresses both transmittance and operational bandwidth simultaneously, which are two critical metrics. The operational bandwidth of this structure in the visible light region is three times that of resonators using dielectric reflectors. This allows for a wider spectral detection range with a single device. Furthermore, compared to resonators made of metallic reflectors, our design offers higher energy utilization efficiency. This means that the long-wavelength region, typically discarded due to lower transmittance, can be fully utilized.

To enhance transmission efficiency and broaden the operational bandwidth of the FP filter, we relied on the rigorous design matrix theory for the metal–dielectric composite film system, involving precise calculations and derivations. The concept of potential transmittance, represented by *ψ* = *T*/(1 − *R*), was introduced by Bering and Turner [36]. Considering *R* + *T* + *A* = 1, it can be deduced that *A* = (1 − *R*)(1 − *ψ*). The concept of potential transmission suggests that in the metal–dielectric composite film system, absorption is dependent on the arrangement of the dielectric films. By designing suitable film stacks on both sides of the metal film, it is possible to somewhat reduce the absorption of the overall film system. This allows for a balance to be struck between reflection, transmission, and absorption. Assuming the addition of multiple layers of dielectric films on both sides of a metal film of fixed thickness, the system can be represented mathematically using the matrix equation for multilayer films. In this equation, *B* and *C* denote the brightness and contrast coefficients, respectively [37].

(3) $$\left[\begin{array}{c} B\\ C \end{array}\right]=\prod_{j=1}^{k}M_{j}\left[\begin{array}{c} 1\\ \eta_{k+1} \end{array}\right]=\left\{\prod_{j=1}^{i-1}\left[\begin{array}{cc} \cos\delta_{j} & \frac{i\sin\delta_{j}}{\eta_{j}}\\ i\eta_{j}\sin\delta_{j} & \cos\delta_{j} \end{array}\right]\right\}M_{j}\left\{\prod_{p=i+1}^{k}\left[\begin{array}{cc} \cos\delta_{p} & \frac{i\sin\delta_{p}}{\eta_{p}}\\ i\eta_{p}\sin\delta_{p} & \cos\delta_{p} \end{array}\right]\right\}\left[\begin{array}{c} 1\\ \eta_{k+1} \end{array}\right]$$

where *M_j_* is the characteristic matrix of the thin film, *δ_j_* and *δ_p_* represent the effective phase thickness of the optical wave in the *j* and *p* thin films, *η_j_* and *η_p_* are the effective optical admittance of the optical wave in the *j* and *p* thin films, and *η_k_*_+1_ is the optical admittance of the substrate (the incident medium is air, so *η*_0_ = 1). For the thin metal film, its characteristic matrix *M_j_* can be expressed as follows:

(4) $$M_{j}=\left[\begin{array}{cc} \cos\frac{2\pi d_{i}(n-ik)}{\lambda } & i\frac{\sin\frac{2\pi d_{i}(n-ik)}{\lambda }}{\left(n-ik\right)}\\ i(n-ik)\sin\frac{2\pi d_{i}(n-ik)}{\lambda } & \cos\frac{2\pi d_{i}(n-ik)}{\lambda }j \end{array}\right]$$

(5) $$Y=\frac{C}{B}$$

(6) $$R=\left(\frac{\eta_{0}-Y}{\eta_{0}+Y}\right){\left(\frac{\eta_{0}-Y}{\eta_{0}+Y}\right)}^{*}$$

(7) $$T=\frac{4\eta_{0}\eta_{k+1}}{(\eta_{0}B+C){\left(\eta_{0}B+C\right)}^{*}}$$

where *d_i_* is the thickness of the metal film, *n* − *ik* is the optical constant of the thin metal film, and *λ* is the wavelength of the incident light. We fixed the thickness of the metal film at 40 nm, and the chosen metal material was silver (*Ag*), which allowed us to establish the matrix *M_j_* as a known and fixed matrix. By substituting Equation (4) into Equation (3), which represents the matrix equation for the multilayer film system, we could determine the equivalent optical admittance of the metal–dielectric composite film system. This is represented in Equation (5). With the result from Equation (5), we could then calculate the reflection of the film system using Equation (6) and the transmission of the film system using Equation (7). This approach allowed us to comprehensively analyze the optical properties of the metal–dielectric composite film system.

(8) $$\left[\begin{array}{c} B\\ C \end{array}\right]=M_{m}M_{d}\left[\begin{array}{c} 1\\ 1 \end{array}\right]=\left[\begin{array}{cc} \cos\frac{2\pi d_{m}(n_{m}-ik_{m})}{\lambda } & i\frac{\sin\frac{2\pi d_{m}(n_{m}-ik_{m})}{\lambda }}{\left(n_{m}-ik_{m}\right)}\\ i(n_{m}-ik_{m})\sin\frac{2\pi d_{m}(n_{m}-ik_{m})}{\lambda } & \cos\frac{2\pi d_{m}(n_{m}-ik_{m})}{\lambda }j \end{array}\right]\left[\begin{array}{cc} \cos\frac{2\pi d_{d}(n_{d}-ik_{d})}{\lambda } & i\frac{\sin\frac{2\pi d_{d}(n_{d}-ik_{d})}{\lambda }}{\left(n_{d}-ik_{d}\right)}\\ i(n_{d}-ik_{d})\sin\frac{2\pi d_{d}(n_{d}-ik_{d})}{\lambda } & \cos\frac{2\pi d_{d}(n_{d}-ik_{d})}{\lambda }j \end{array}\right]\left[\begin{array}{c} 1\\ 1 \end{array}\right]$$

(9) $$T_{\max}=\frac{4(\frac{B-C}{B+C}){\left(\frac{B-C}{B+C}\right)}^{*}}{(B+C){\left(B+C\right)}^{*}}$$

For a medium with a refractive index *n* – *ik*, its characteristic equation can also be represented by Equation (4). We assumed the addition of a dielectric film on one side of the metal film, and the matrix equation for this bilayer film system could be expressed as Equation (8), where *M_m_* represents the characteristic matrix of the metal film and *M_d_* represents the characteristic matrix of the dielectric film. By computing Equation (8), we could obtain expressions for *B* and *C* for the metal–dielectric film system. We then combined Equation (1) with Equations (5)–(7) to obtain Equation (9). To achieve high transmittance, we fixed the metal film layer to be a 40 nm thick metallic silver layer, specified the dielectric material as TiO_2_, and substituted these known parameters into Equations (8) and (9). If *T*_max_ is set to be greater than or equal to 0.5, then this equation becomes a single unknown equation containing only the thickness of the dielectric film layer. Solving this equation yields an optimal dielectric film layer thickness of approximately 68 nm. To validate our computational results, we conducted simulation experiments using the Ansys Lumerical FDTD (www.lumerical.com) to investigate how changes in each parameter affect device performance.

**Figure 3:**
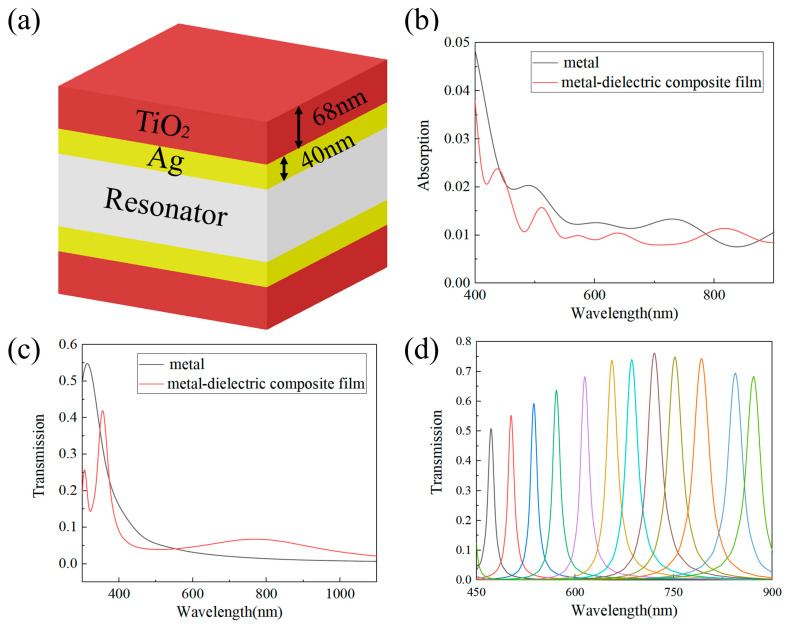
(**a**) Schematic diagram of the metal–dielectric-composite-film-based FP filter structure; (**b**) comparison of the absorption rates between the metal–dielectric composite film system and the metal thin film; (**c**) comparison of the transmission rates between the metal–dielectric composite film system and the metal film system; (**d**) transmission spectra for 12 spectral channels.

Compared to the thin metal film, the metal–dielectric composite film system shows enhanced transmission in the 570–1000 nm range and a decrease in absorption in the 400–800 nm range (Figure 3b,c). These improvements suggest that the Fabry–Pérot (FP) filter, which uses the metal–dielectric composite film system as a reflective mirror, would exhibit superior transmission characteristics. To ensure a single transmission peak within the operational bandwidth, a working wavelength range of 450–900 nm was carefully selected. Simulation results indicate that within this 450 nm wide operational bandwidth, the transmission peak of all channels exceeds 50%, with the full width at half maximum (FWHM) ranging between 10 nm and 25 nm (Figure 2c). In contrast, resonator cavities composed of a metallic film of equivalent thickness exhibit an average peak transmittance below 40% within the same wavelength range. Upon comparing the Fabry–Pérot (FP) filters constructed using the metal–dielectric composite film system with those solely employing a 40 nm thick metal film as a reflective mirror, we observed significant differences. We selected seven cavity lengths within the range of 116–258 nm for the resonant cavity and computed the transmission spectra of both the metal resonant cavity and the metal–dielectric composite film cavity corresponding to these lengths. It became evident that the transmission peak of the metal–dielectric composite film system filter surpassed that of the latter in both the visible and near-infrared regions (Figure 2b,c). This finding underscores the superior optical performance of the metal–dielectric composite film system, rendering it a more advantageous solution for broadband spectral imaging.

### 2.2. Design of Polarization Filter

To enable simultaneous functionality in a single device, the design of a polarization filter structure must adhere to the principles of planarization and integration. Subwavelength metal gratings, known for their exceptional planarization capabilities, can effectively filter the incident light of various polarization states. When the electric field component parallel to the grating direction (TE light) enters the subwavelength metal grating, it triggers the excitation of electrons within the grating. This results in free oscillation along the grating direction, thereby preventing reflection from the grating [37]. On the other hand, the electric field component perpendicular to the grating direction (TM light) undergoes partial absorption and successfully passes through the grating. We employed the Effective Medium Theory (EMT) to infer periodic grating structures. This approach simplifies the model calculation process by treating the metal grating as a uniform medium model. The effective refractive indices for TE and TM-polarized light within the grating are expressed as follows [38,39,40]:

(10) $$n_{TE}=\sqrt{f{\left(n_{1}+ik_{1}\right)}^{2}+(1-f){\left(n_{2}+ik_{2}\right)}^{2}}$$

(11) $$n_{TM}=\sqrt{\frac{{\left(n_{1}+ik_{1}\right)}^{2}+{\left(n_{2}+ik_{2}\right)}^{2}}{f{\left(n_{2}+ik_{2}\right)}^{2}+(1-f){\left(n_{1}+ik_{1}\right)}^{2}}}$$

(12) $$ER=10\mathrm{lg}\left(\frac{T_{TM}}{T_{TE}}\right)$$

**Figure 4:**
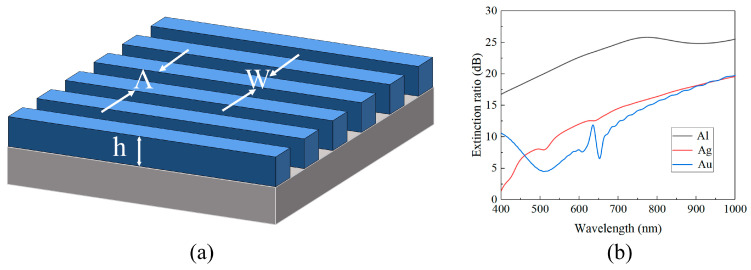
(**a**) Schematic diagram of subwavelength metal grating structure with *Λ* = 200 nm, *h* = 100 nm, and *f* = 50% and (**b**) extinction ratio for different metal materials under the structure in (**a**).

Here, *n* represents the material refractive index, *f* denotes the grating duty cycle (*W*/*Λ*), and *k* is the extinction coefficient (Figure 4a). The calculation formula for the extinction ratio is shown in Equation (12). By substituting Equations (10) and (11) into Equation (7), we can ascertain that, when the duty cycle and grating height are fixed, the transmittance of the grating structure solely depends on the material’s intrinsic parameters, determined by the effective media *n* and *k*. According to the calculation formula for the extinction ratio (Equation (12)), the extinction ratio (*ER*) is related to the transmittance at the corresponding wavelength. Therefore, the extinction ratio is only dependent on *n* and *k*. Taking into consideration the fixed grating structure with *Λ* = 200 nm, *h* = 100 nm, and *f* = 0.5, and only varying the material of the grating structure, the simulation results are depicted in Figure 4b. Based on the extinction ratio curves of the three different materials shown in the figure, we identified aluminum (Al) as the material possessing a superior extinction ratio in the 400–1000 nm wavelength range.

(13) $$\Lambda =\frac{k\lambda }{n_{0}\sin\theta +n}(k=1,2,3\ldots )$$

**Figure 5:**
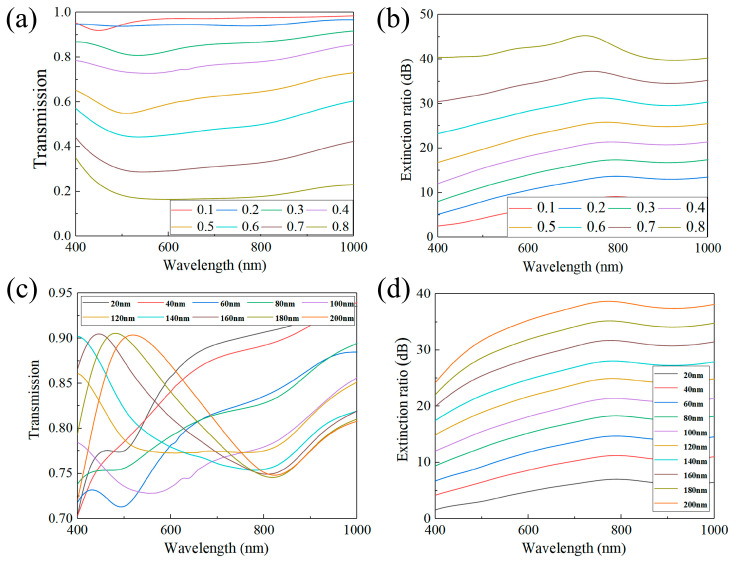
(**a**) Transmission of TM light corresponding to different *f*; (**b**) extinction ratio corresponding to different *f*; (**c**) transmittance of TM light corresponding to different *h*; (**d**) extinction ratio corresponding to different *h*.

Subwavelength gratings, with a period below the incident wavelength order, ensure that higher-order diffraction waves beyond the 0th order are evanescent waves devoid of energy. To mitigate the Raman anomalous effect caused by the grating period approaching the incident wavelength, a minimum operating wavelength of 400 nm was set. After applying this to the Raman anomalous critical period calculation formula (Equation (13)), where *Λ* is the grating period, *θ* is the incident angle of light, and *n*_0_ and *n* are the refractive indices of the incident medium (air, *n*_0_ = 1) and the substrate medium (SiO_2_, *n* = 1.45), respectively, the critical period was calculated based on Equation (6) and the results confirmed it to be 274 nm. To circumvent this critical period, the grating period was intentionally set at 200 nm. We calculated the effects of varying duty cycles (*f*) on *T*_TM_ and *ER* in the high reflection region of the metal–dielectric composite film (as shown in Figure 5a,b). The findings revealed that as the duty cycle increased from 0.1 to 0.8, the transmittance of TM light decreased, while the extinction ratio increased. Recognizing that the filtering units in multifunctional devices can attenuate some incident light, we designed the polarization grating to maintain higher transmittance for TM light. This led us to choose a duty cycle of 0.4. Under these conditions, the average transmittance reached 75% within the 400–1000 nm wavelength range, and the max. extinction ratio was 21.7 dB.

With a fixed duty cycle of 0.4, determining the grating height parameter was essential. Calculations assessing the transmittance and extinction ratio of TM-polarized light at various heights were performed (refer to Figure 5c,d). Our results indicated that a grating height exceeding 150 nm ensured both good average transmittance and a high extinction ratio. Consequently, the grating height was set to 160 nm.

To bestow a specific stacking property on the polarization functional layer, the voids in the metal grating were filled with PMMA. In addition, a layer of PMMA film, with a predetermined thickness, was designed to sit above the grating. With the introduction of PMMA material and the filling of the aluminum grating structure’s gaps, the periodic arrangement created by PMMA and aluminum could be viewed as a new equivalent medium layer. This medium layer could then be treated as a thin film with a thickness of 160 nm and an effective refractive index of *n_al-PMMA_*. Simultaneously, the PMMA film layer above it, with a specified thickness, acted as an extra dielectric layer. This contributed to the formation of a unique film system in combination with the grating’s equivalent medium layer. In this film system, the parameters of the underlying grating’s equivalent medium layer remained unchanged, with only the thickness of the PMMA layer affecting the system’s operational characteristics.

**Figure 6:**
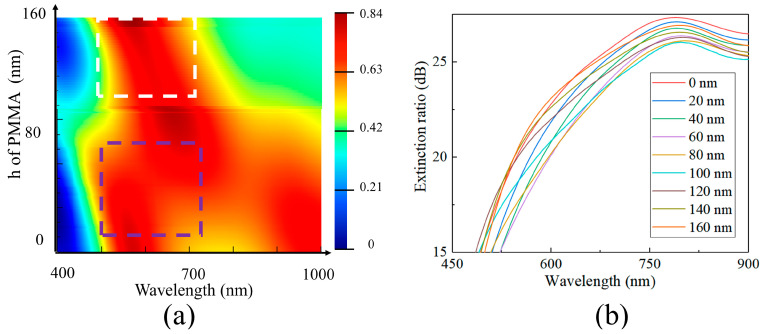
(**a**) Transmittance corresponding to different PMMA height values and (**b**) extinction ratio corresponding to different PMMA height values.

We also performed calculations by scanning the thickness of the PMMA layer within the range of 0–160 nm to evaluate the transmittance of the grating film system for TM light (Figure 6a). For the grating film system, when the PMMA layer is less than 100 nm (as indicated by the blue dashed box in Figure 6a), the system displays a wider transmission band. However, the region with transmittance above 60% is narrow and discontinuous. In contrast, when the PMMA layer’s thickness exceeds 100 nm (as indicated by the white dashed box in Figure 6a), the system exhibits a broader and more stable high-transmission band. Interestingly, the thickness of the PMMA layer has a minimal effect on the extinction ratio of the polarization film system (Figure 6b). To ensure a high energy utilization rate for individual device layers, we meticulously set the thickness of the PMMA layer to 120 nm. At this specific thickness, the average transmittance of the polarization layer for TM light in the 500–700 nm wavelength range exceeds 75%.

### 2.3. Design of Spectral Polarization Multifunctional Device

**Figure 7:**
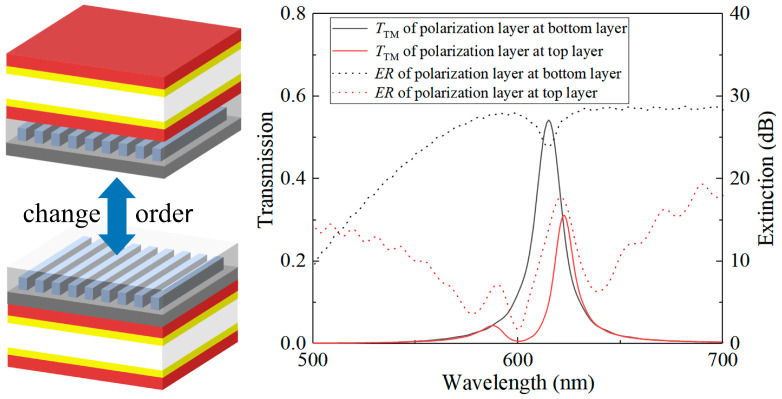
Alterations in the stacking sequence of the resonant cavity and polarization devices yield distinct outcomes.

The polarization device layer, which comprises a metal grating and PMMA, maintains a uniform thickness to form a consistently thin film layer with effective planarization. This layer is part of a multi-channel resonant cavity structure created by the metal–dielectric composite film. By adjusting the length of the resonant cavity, the structure can modify channel positions, leading to distinct heights for the filtering structures in different channels. This strategic positioning of the polarization layer at the bottom simplifies the device’s processing complexity. We conducted comprehensive calculations to assess the impact of various stacking sequences of the polarization layer and the resonant cavity layer on the multifunctional device’s overall performance. We calculated the transmittance spectra of incident light passing through the resonant cavity both before and after the introduction of the polarization layer independently. As depicted in Figure 7, the results indicate that when the polarization layer is positioned at the bottom, the overall structure displays higher transmittance and a superior extinction ratio.

From a practical feasibility standpoint, it is more achievable in real-world processing to place the polarization layer at the bottom with uniform height. Hence, in a single device unit designed for simultaneous spectral polarization detection, the polarization device is situated at the bottom, with the filtering device positioned above it. This configuration allows incident light traversing the multifunctional device to first experience resonance in the cavity, leading to a transmission peak curve. Following this, the light proceeds through the polarization device. This step enables the identification of the incident light’s polarization state, thereby facilitating the detection of both the spectral and polarization information of the target.

## 3. Result and Discussions

**Figure 8:**
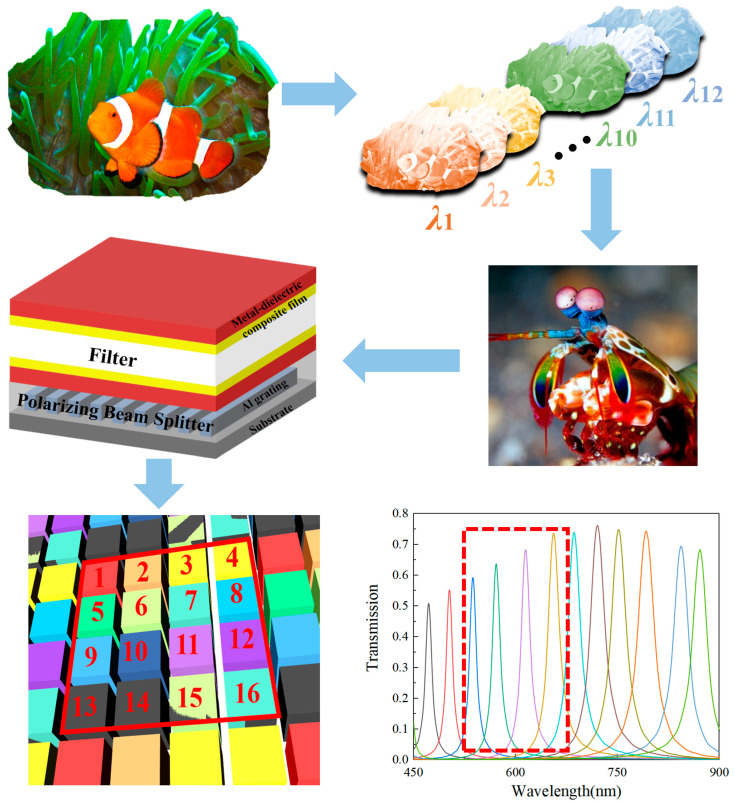
Biomimetic mantis shrimp visual system design schematic and transmittance spectra of the 12 spectral channels (The red dotted boxes are the spectral channels corresponding to the four selected multi-functional devices).

Our multifunctional device, inspired by the complex visual system of the mantis shrimp, which boasts a working bandwidth of 430 nm and includes 16 types of photoreceptors, operates within an expansive 450–900 nm working bandwidth. We have configured 12 spectral channels within this device to achieve multispectral color perception (as shown in Figure 8). Table 1 provides an in-depth breakdown of the design parameters for these 12 spectral channels. Each channel is meticulously tailored with filters at specific heights. Moreover, guided by computational analyses, we strategically identified four spectral channels within the 500–700 nm range in the polarization device layer (highlighted within the red dashed box in Figure 8). These channels were chosen due to their heightened transmittance. These specific channels were deliberately selected to serve as pathways for achieving spectral polarization functionalities. This thoughtful integration resulted in the creation of a 16-channel multifunctional device array. This array encompasses the initial 12 spectral channels, supplemented by an additional four channels specifically engineered for spectral polarization versatility. The multifunctional device array convincingly demonstrates its ability to concurrently accommodate a range of functionalities within the broad 450–900 nm working bandwidth. Notably, the design exhibits adaptability for processing on CMOS devices, underscoring its practical viability for implementation.

**Figure 9:**
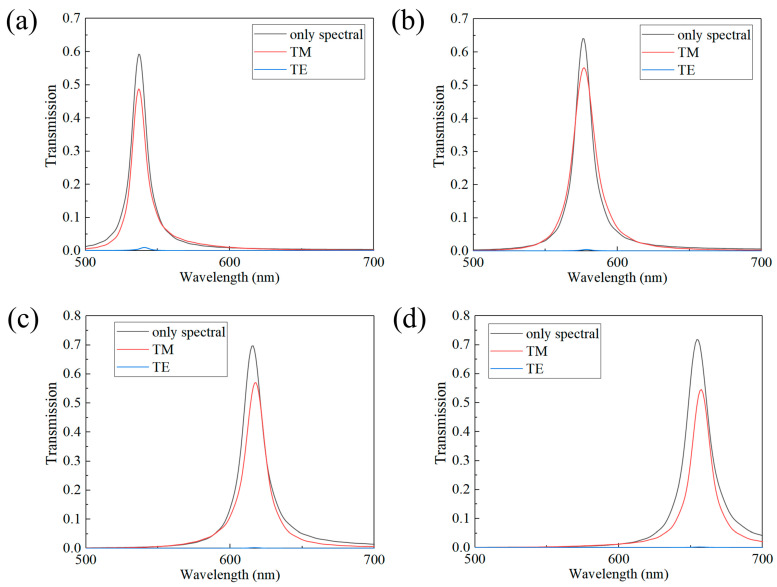
Transmittance spectra of different channels with and without the addition of polarization structures: (**a**) central wavelength at 537 nm; (**b**) central wavelength at 571 nm; (**c**) central wavelength at 614 nm; (**d**) central wavelength at 655 nm.

We carried out calculations to examine the transmission peaks produced when TM and TE light separately impinged on the four multifunctional device channels that incorporated polarization layers. These results were then contrasted with those from channels that lacked polarization structures, serving as a reference. As illustrated in Figure 9, the findings indicate that the incorporation of the polarization layer in the four multifunctional device channels results in effective filtering for TE light. Despite the inevitable absorption effects of the metal grating on TM light, which cause further attenuation of the incident light energy, careful optimization of device parameters enables the peak transmittance of TM light to be sustained at around 50%.

(14) $$S=\left[\begin{array}{c} S0\\ S1\\ S2\\ S3 \end{array}\right]=\left[\begin{array}{c} E_{x}^{2}+E_{y}^{2}\\ E_{x}^{2}-E_{y}^{2}\\ 2E_{x}E_{y}\cos\delta \\ 2E_{x}E_{y}\sin\delta \end{array}\right]$$

(15) $$P=\frac{\sqrt{{S_{1}}^{2}+{S_{2}}^{2}+{S_{3}}^{2}}}{S_{0}}$$

To quantitatively evaluate the polarization states, we utilized the Stokes matrix (Equation (14)) and calculated the degree of polarization *P* using Equation (15) to determine the polarization state of the incident light, generally with 1 ≥ *P* ≥ 0. The Stokes matrix is represented by Equation (14), where *Ex* and *Ey* denote the components of the light wave, and *δ* represents the phase difference between the two components. When *P* = 0, the light wave is completely unpolarized, i.e., natural light. When 1 > *P* > 0, the light wave is partially polarized, and when *P* = 1, the light wave is completely polarized. Division-of-focal-plane (DoFP) polarization imaging sensors are typically composed of 2 × 2 metal grating pixels to simultaneously capture intensity information for different polarization states in real time [41].

**Figure 10:**
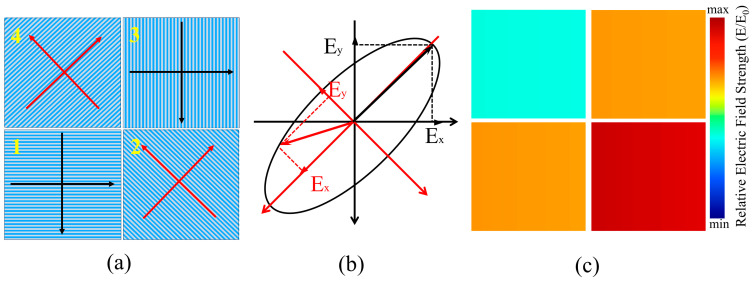
(**a**) Schematic diagram of the grating arrangement in DoFP; (**b**) decomposition diagram of elliptically polarized light after entering DoFP; (**c**) relative electric field intensity distribution of elliptically polarized light after passing through DoFP.

We also employed a 2 × 2 polarization structure array, where each polarization device consisted of metal gratings oriented at 0°, 45°, 90°, and 135°, corresponding to the numbered regions 1, 2, 3, and 4 in the diagram (Figure 10a). By collecting intensity information within the four polarization sub-channels, *Ex*, *Ey*, and Stokes parameters could be determined. Regarding polarized light, both linearly polarized light and circularly polarized light can be considered as special forms of elliptically polarized light, with expressions such as $\vec{E}=A_{x}\cos(\omega t-kz)\vec{i}+A_{y}\cos(\omega t-kz+\Delta \phi )\vec{j}$, where *A* represents amplitude and ∆*φ* represents the phase difference. We could decompose it in the orthogonal coordinate system as ${\vec{E}}_{x}=A_{x}\cos(\omega t-kz)\vec{i}$, $\vec{E_{y}}=A_{y}\cos(\omega t-kz+\Delta \phi )\vec{j}$. In the case of linearly polarized light, *Ex* or *Ey* equals zero, while for circularly polarized light, *Ex* = *Ey*.

The four distinct grating structures can be interpreted as two sets of coordinate systems, with a 45° angle separating them (as shown in Figure 9a). When elliptically polarized light, illustrated in Figure 10b, strikes the DoFP, and only polarization gratings from groups 2 and 4 (which are mutually perpendicular) are present, two signals of equal intensity will be produced, as shown in Figure 10c. This result, which aligns with the findings for circularly polarized light, prevents the accurate determination of the incident light’s polarization state. To accurately identify the polarization state of the incident light, it becomes necessary to decompose the incident light into these two sets of coordinate systems.

**Figure 11:**
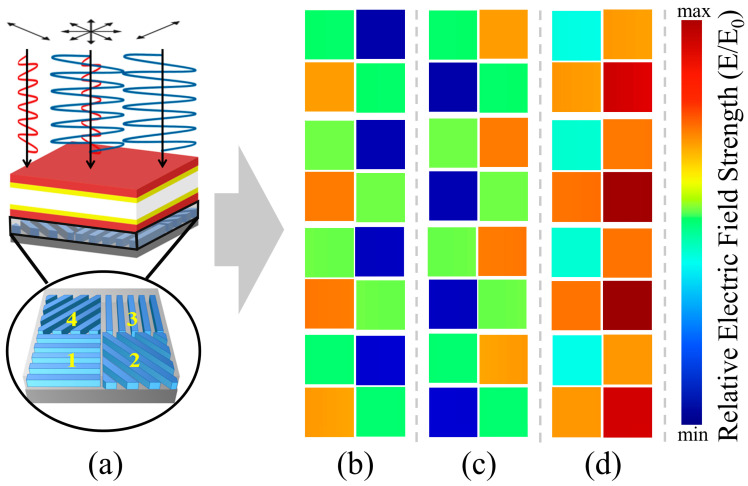
(**a**) Schematic illustration of the multifunctional channels and division-of-focal-plane (DoFP) structure, and distribution of the relative electric field intensity for incident light with different polarization states after passing through the four multifunctional channels: (**b**) TM-polarized light; (**c**) TE-polarized light; and (**d**) elliptically polarized light.

We introduced TM light, TE light, and elliptically polarized light into the four chosen multifunctional channels, each with central wavelengths at 537 nm, 571 nm, 614 nm, and 655 nm, respectively. Through computational simulations, we analyzed their relative electric field intensity distributions (*E*/*E*_0_) under various incident light conditions. As depicted in Figure 11, it is clear that the DoFP device, located beneath the multifunctional device, generates distinct responses to incident light with differing polarization states. By incorporating the signal intensities received in the 1–2–3–4 sub-channels into the Stokes matrix calculations, we could effectively determine the polarization state of the incident light. In real-world applications, where the multifunctional device is seamlessly integrated with CMOS devices, the received signal intensity requires additional algorithmic data processing and computation. This step is vital for accurately reconstructing the more complex polarization states of the light source.

The multispectral polarization multifunctional device array that we designed, when integrated with CMOS devices, ensures miniaturization while simultaneously achieving multiple functionalities. The success of our design is rooted in the careful engineering of a dielectric–metal hybrid film, which enables an impressive working bandwidth of 450 nm. This performance outshines both metal and dielectric resonant cavities, underscoring the versatility of our multifunctional device. A key strength of the designed multispectral polarization multifunctional device array is our ability to achieve a customized central wavelength position within the working bandwidth. This adaptability is achieved through precise adjustments to the thickness of the resonant cavity, enabling us to cater to specific requirements for a variety of applications. By manipulating device parameters, we can finely control the position of multifunctional channels within any region of the 450 nm working bandwidth. While addressing the inevitable broadening of the working bandwidth, which leads to an increase in channel FWHM and a reduction in spectral resolution, we adjusted the thickness of the metal film in the dielectric–metal hybrid structure. This optimization process required a delicate balance between reducing the full width at half maximum (FWHM) and maintaining transmittance. To ensure optimal energy utilization, we carefully selected an optimal thickness for the metal film. Despite these considerations, our multifunctional device demonstrates exceptional overall performance in terms of working bandwidth and transmittance. Achieving multiple functionalities simultaneously presents challenges, especially in preserving the design integrity of filtering and polarization devices during sequential processing. The rapidly evolving landscape of novel devices introduces new possibilities, such as the use of liquid crystal materials to recognize different polarization states of incident light. The introduction of a liquid crystal layer could alleviate alignment difficulties and enable higher energy utilization, allowing for real-time recognition of controllable polarization state patterns [42]. With the rise of planar optical devices, the importance of multifunctional devices designed through various combinations or stacking of different functional devices has become evident. However, the inherent differences in absorption between on-chip metal structures and silicon photonic structures in the visible light range result in decreased energy utilization. Furthermore, the combination and stacking of multifunctional devices may lead to even greater energy losses, presenting a significant challenge for future on-chip device development. Addressing these challenges will be pivotal for the continual advancement of on-chip multifunctional devices.

## 4. Conclusions

In this study, we have introduced an innovative design inspired by the mantis shrimp’s multifaceted visual system. By emulating the bio-inspired visual camera of the mantis shrimp, we have developed a device that combines a broadband metal–dielectric-composite-film-based Fabry–Pérot (FP) filter array with a polarization detection array. This amalgamation offers comprehensive optical capabilities within a single device, demonstrating the potential for seamless integration into complementary metal–oxide–semiconductor (CMOS) devices. Guided by simulation results, we meticulously optimized device parameters, including bandwidth and spectral resolution, to develop a 16-channel filter device. This device significantly expands the operational bandwidth, covering the range of 450–900 nm. This enhancement was achieved by incorporating a high-refractive-index matching film on the metal film. Furthermore, to enhance our device’s ability to distinguish between different polarization states in four selected spectral channels, we integrated an aluminum grating array beneath the FP filtering structures. This inclusion of polarization detection functionality broadens the potential applications of our device across diverse scenarios. Inspired by bio-mimicry, our device adeptly integrates multiple functionalities into a cohesive unit, providing a fresh perspective on on-chip spectral imaging. The proposed design has promising prospects for future applications in underwater imaging, mobile target detection, and recognition, as well as tumor detection. The successful fusion of spectral and polarization detection in a single device marks a significant advancement in optical sensor technology, paving the way for enhanced applications in various fields. Our multifunctional device leverages bio-inspired design principles and addresses practical challenges associated with on-chip integration. As we look forward, further refinements and advancements in on-chip multifunctional devices will continue to drive innovation in optical sensing, paving the way for enhanced applications in various fields.

## Figures and Tables

**Table 1:** Detailed design parameters of 12 spectral channels.

	Channel 1	Channel 2	Channel 3	Channel 4	Channel 5	Channel 6	Channel 7	Channel 8	Channel 9	Channel 10	Channel 11	Channel 12
Thickness of the resonator (nm)	112	124	136	150	164	178	190	200	212	224	242	250
Wavelength (nm)	472	502	537	571	614	655	686	719	752	792	843	871

## Data Availability

Data are contained within the article.
